# Stress reactivity elicits a tissue-specific reduction in telomere length in aging zebrafish (*Danio rerio*)

**DOI:** 10.1038/s41598-020-79615-1

**Published:** 2021-01-11

**Authors:** James R. Evans, Jose V. Torres-Pérez, Maria Elena Miletto Petrazzini, Riva Riley, Caroline H. Brennan

**Affiliations:** 1 School of Biological and Chemical Sciences, Queen Mary University of London, Mile End Rd, London, E1 4NS UK; 2 Department of Biomedical Sciences, University of Padova, Via Ugo Bassi 58/B, 35131 Padova, Italy

**Keywords:** Ageing, Cardiovascular biology, Neural ageing

## Abstract

Individual differences in personality are associated with variation in healthy aging. Health behaviours are often cited as the likely explanation for this association; however, an underlying biological mechanism may also exist. Accelerated leukocyte telomere shortening is implicated in multiple age-related diseases and is associated with chronic activation of the hypothalamus–pituitary–adrenal (HPA) axis, providing a link between stress-related personality differences and adverse health outcomes. However, the effects of the HPA axis are tissue specific. Thus, leukocyte telomere length may not accurately reflect telomere length in disease-relevant tissues. Here, we examined the correlation between stress reactivity and telomere length in heart and brain tissue in young (6–9 month) and aging (18 month) zebrafish. Stress reactivity was assessed by tank diving and through gene expression. Telomere length was assessed using quantitative PCR. We show that aging zebrafish have shorter telomeres in both heart and brain. Telomere length was inversely related to stress reactivity in heart but not brain of aging individuals. These data support the hypotheses that an anxious predisposition contributes to accelerated telomere shortening in heart tissue, which may have important implications for our understanding of age-related heart disease, and that stress reactivity contributes to age-related telomere shortening in a tissue-specific manner.

## Introduction

Multiple epidemiological studies report associations between personality traits such as dispositional optimism, anxiety or depression and (un)healthy aging in both cross-sectional and longitudinal designs^1,2^. However, the majority of studies of personality and aging have relied on self-report measures of symptoms, pain or physical functioning in old age. This means that much of the correlation between personality and healthy aging may not be causal, but instead could be explained by the reporting biases of individuals. Recognising this limitation, some studies have focused on objective measures of aging including mortality, cardiovascular disease, immune function, or cancer. While these studies support the role of personality in healthy aging^2^, rather than measuring aging per se, many of these objective measures relate to symptoms of diseases or disorders with their own specific genetic and environmental risk factors.

In an attempt to obtain a more objective measure of the impact of personality on aging, more recent research has examined the link between personality and telomere length^3–6^. Telomeres, tandem repeat guanine-rich DNA sequences, are specialised protective caps located at the ends of eukaryotic chromosomes. Crucial for the maintenance of genomic stability, telomeres protect against the attrition of genetic material, shortening with each cell division in most somatic tissues due to incomplete chromosome replication^7^. Over time, progressively shortened telomeres may lead to cell-cycle arrest^8^, apoptosis^9^ or senescence^10^. As such, telomere length may be considered as both a ‘mitotic clock’, reflecting cellular aging, and a mechanism through which age-related disease occurs^11,12^. Using leukocyte telomere length as a biomarker of cellular aging, several studies have reported a significant correlation between personality traits and telomere length^3–6^.

Whilst personality influences several behaviours that may contribute to accelerated cellular aging, for example, exercise, smoking, and dietary habits^13,14^, personality differences, such as optimism or depressive tendency, are also associated with differences in stress perception and physiological stress response^15,16^. Psychological stress leads to activation of the hypothalamus–pituitary–adrenal (HPA) axis, the body’s physiological stress response system. Research has demonstrated a link between physiological stress response and cellular aging, with telomere shortening associated with chronic activation of the HPA axis^17^, as well as with pro-inflammatory cytokines generated during stress related disorders such as major depression^18,19^. Consistent with the hypothesis that individuals with an increased tendency to perceive a given situation as stressful are likely to have a higher lifetime of perceived stress, greater lifetime activation of the HPA axis and age-related disease, the likelihood of onset and progression of age-related diseases is associated with an anxious personality^20–22^ and HPA axis dysfunction^23,24^. It is therefore thought that individual differences in stress reactivity (individual differences in tendency to perceive and respond to a given situation as stressful) may contribute to telomere shortening, i.e. accelerated aging, and, in turn, age-related disease^25–28^.

However, many clinical studies pointing to a relationship between telomere length and either personality or age-related disease assess telomere length from blood samples, reporting leukocyte telomere length as a proxy measure for cellular aging^3–6,29–31^. The true meaning of such research relies upon the fundamental assumption that changes in telomere length are robust across tissues, and that leukocyte telomere length faithfully reflects tissues relevant to disease, such as the heart and brain. However, the HPA axis stress response has been shown to elicit its effects in a tissue-specific manner^32,33^. Thus, leukocyte telomere length may not accurately reflect the relationship between stress reactivity and telomere length in disease-relevant tissues.

Here, we used a population of zebrafish and a pseudo-longitudinal design to test the hypothesis that individual differences in stress reactivity contribute to age-dependent telomere shortening in disease-relevant tissues. Zebrafish have emerged as a promising animal model to explore telomere dynamics due to important similarities with humans. These include high genomic conservation^34^, a hypothalamic-pituitary-interrenal (HPI) axis analogous to the human HPA axis^35^, age-dependent telomere shortening^36–38^, and, unlike mice, similar telomere lengths to humans^39,40^. Furthermore, zebrafish have been shown to have stable individual differences in anxiety-like behaviours and stress reactivity^41^. On the basis that zebrafish with a higher reactivity to a novel stressor are likely fish that have a higher lifetime of perceived stress, and therefore higher lifetime HPI activity, we predict that fish that show high levels of anxiety-like behaviour in a novel tank diving test will have shorter telomeres.

## Materials and methods

### Subjects

All in vivo experimental protocols in this study were reviewed and approved by the QMUL ethics committee (AWERB) following consultation of the ARRIVE guidelines (NC3Rs, UK) and conducted in accordance with the Animals (Scientific Procedures) Act, 1986 and Home Office Licenses. 256 adult Tuebingen (TU, wild type) zebrafish (*Danio rerio*), 134 males and 122 females, were reared to 6–9 months (133 subjects) or 18 months (123 subjects) of age. Fish were housed in a recirculating system (Techniplast, UK) with a light:dark cycle of 14:10 and a constant temperature of 28.4 °C. All subjects were fed twice daily with ZM-400 fry food (Zebrafish Management Ltd, Winchester, United Kingdom) in the morning and brine shrimp in the afternoon.

### Study design

To test the hypothesis that stress reactivity contributes to age-related telomere shortening in a tissue-specific manner we identified individual differences in the zebrafish anxiety-like behavioural response in a population of adult zebrafish at 6–9 months (young) and 18 months (aging) of age using the novel tank diving assay. The novel tank diving assay is a zebrafish version of the rodent open field test and therefore assesses anxiety-like behavioural response to an immediate stressor; i.e. stress reactivity. Behaviourally determined stress reactivity was validated through the mRNA expression of HPI axis related genes. Correlations between behaviourally determined stress reactivity, HPI axis gene expression and tissue-specific telomere length in young and old, male and female zebrafish were determined.

### Novel tank diving assay

**Figure 1:**
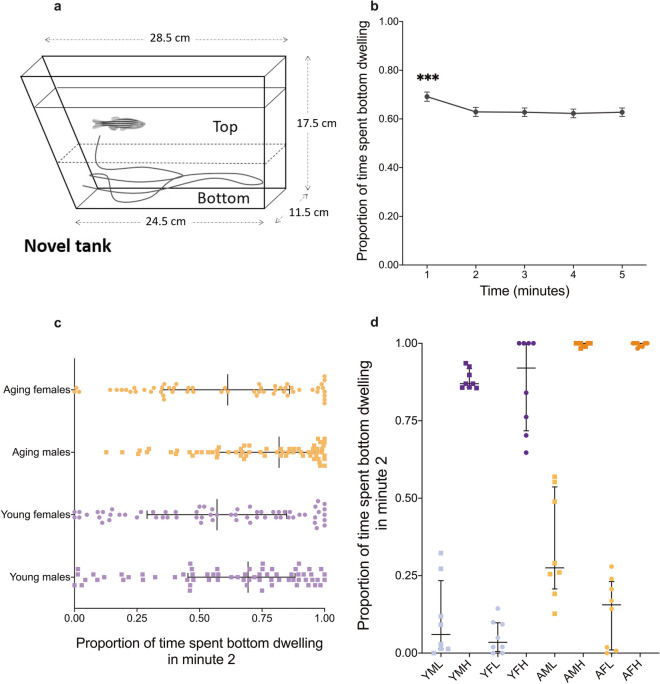
Zebrafish selected for molecular analysis based on behaviourally determined stress reactivity. (**a**) Diagram of the novel tank assay: each fish is individually transferred from its home tank to a novel test tank. The time (seconds) spent in the bottom in each minute was used for analysis. (**b**) Entire population results for bottom dwelling by minute over the course of the novel tank test. (**c**) Population distribution of young (6–9 month) and aging (18 month) males and females based on their bottom dwelling in minute two (*N* = 256). (**d**) Bottom dwelling differences in minute two for the fish selected for molecular analysis (*N* = 64). Note that (**d**) corresponds to either extreme of the population distribution in (**c**). Error bars in (**b**) represent ± standard error mean (SEM). For (**c**) and (**d**) middle lines indicate median values and error bars represent interquartile range. *YFL* young female low stress reactivity, *YFH* young female high stress reactivity, *YML* young male low stress reactivity, *YMH* young male high stress reactivity, *AFL* aging female low stress reactivity, *AFH* aging female high stress reactivity, *AML* aging male low stress reactivity, *AMH* aging male high stress reactivity. ***p-value < 0.001.

The novel tank diving test was performed as described previously^34^. Briefly, after one hour of acclimation to the test room, zebrafish were individually placed in a 1.5-L trapezoid tank filled with system water (Fig. 1a). Their swimming behaviour was immediately recorded from the side using a DMK 21AF04 Firewire Monochrome camera for 5 min. Swimming activity, including the parameters of bottom dwelling, mobility state and distance covered, were tracked and analysed using EthoVision software (Noldus Information Technology, Wageningen, NL). Water was replaced between trials. Zebrafish were tested in two separate batches. For each batch the test was conducted over consecutive days between 10:00 and 13:00, with groups counter-balanced for age. On completion of the assay, subjects were single housed until assigned stress reactivity status and/or tissue harvesting. We performed a Tukey posthoc test on the bottom dwelling data pooled across age and sex to determine which time point represented the earliest ‘recovery’ from the stress. We constructed a betabinomial GLMM based on this pooled data with the proportion of time spent swimming on the bottom as the response variable, minute as the fixed effect, and batch number and individual ID as random effects. The determined ‘earliest recovery’ time point was used to select the individuals for molecular analysis.

### Tissue harvesting

Fish used for dissection were selected based on bottom dwelling results (Fig. 1b). Fish were sorted as high/low stress reactive based on their bottom dwelling time in minute 2 (Fig. 1c). We selected eight fish, as a conservative estimate of the minimum number likely to yield signficant results based on results from previous studies^37^, from the top and bottom of the distribution for each age and sex (N = 64) (Fig. 1d). The fish were euthanised with overdose of tricaine methane sulfonate (250 mg/L, MS222, sigma). Samples of whole-brain and heart tissue were dissected out, immediately snap-frozen and stored at − 80 °C until required.

### Extracting genomic DNA

Genomic DNA was isolated from whole-brain and heart samples using the HotSHOT protocol^42^. In summary, 100 μL of 50 mM NaOH was added to each sample, which was then incubated at 95 °C for 30 min in a heat block. Once completed, 10 μL of 1 M Tris–HCL, pH 8.0, was added to neutralise the pH.

### Extracting mRNA and cDNA library generation

Total RNA was isolated from whole-brain samples using TRIzol. The protocol followed is as stated in the TRIzol Reagent User Guide (Invitrogen). Briefly, following sample homogenisation with TRIzol, RNA was isolated through precipitation, washing, and resuspension. RNA concentrations and quality were checked using a Thermo Scientific NanoDrop 2000. Corresponding cDNA libraries were generated using the ProtoScript II First Strand cDNA Synthesis Kit (NEB (UK) Ltd., Hitchin, UK).

### Real-time quantitative PCR

In order to investigate differences in HPI axis and *smurf2* gene expression, relative qPCR assays were then performed using SYBR Green (Applied Biosystems) and the CFX Connect Real-Time System (Bio-Rad), with all reactions performed in triplicate. Reference genes for these qPCR analyses were *β-actin* and *rpll3α*. PCR reactions were performed at 95 °C for 5 min followed by 50 cycles of 95 °C for 10 s, 60 °C for 12 s and 72 °C for 12 s. Relative mRNA expression was calculated using a modified version of the Pffafl method to account for multiple reference genes and slight variation in primer amplification efficiency^43^ (Supplementary Table 4). All primer sequences can be found in Supplementary Table 4. Missing data were assigned the highest measured Cq for that gene of interest plus one as performed previously^44^.

To analyse relative average telomere length of the samples, qPCR was again conducted using SYBR Green (Applied Biosystems) and the CFX Connect Real-Time System (Bio-Rad), with all reactions performed in triplicate. The protocol used was a modified version of the method described by Cawthon^45^. Telomere length qPCR reactions were performed at 95 °C for 10 min followed by 40 cycles of 95 °C for 15 s and 54 °C for 2 min. Relative telomere length was expressed as 1/(Cq_telomere_/Cq_SingleCopyGene_). The single copy gene used as reference for the relative ratio was *c-fos*, primers without intra-exon introns (Supplementary Table 4).

### Statistical analysis

All statistical analyses of behavioural data were carried out in R version 3.2.2 (R core developer team), and linear mixed effects models (LME) were fitted using the lme4 package^46^, generalized mixed effect models (GLMM) were fitted using the glmmTMB package^47^. Data distributions were initially assessed visually, and model diagnostics were subsequently checked to assure appropriate fits. We hence fitted LMEs for normally distributed data (total distance moved) and GLMMs with betabinomial error distributions where binomial models had been overdispersed^48^. We used the emmeans package in R (Lenth, 2019) to perform Tukey posthoc tests. Unless indicated, the details of our posthoc analysis are contained in our Supplementary Materials.

Differences in gene expression and telomere length were assessed using three-way between-subjects ANOVAs with age (aging, young), sex (male, female), and stress reactivity (low, high) as the independent variables. All follow-up tests generating simple effect comparisons were conducted with the use of SPSS syntax and, to account for multiple testing, Bonferroni corrections were applied. Pearson’s product-moment correlations were run to assess the relationship between telomere length, HPI axis gene expression and *smurf2* gene expression. Normal distribution of variables was assessed by Shapiro–Wilk’s test (*p* > 0.05). Gene expression data were log 10 transformed to achieve normal distribution for parametric statistical analysis. Gene expression descriptive statistics for significant effects and interactions are shown as log 10 transformed. Log 10 transformed descriptive statistics are presented in Supplementary Table 2 for three-way between-subjects ANOVAs. Untransformed relative fold gene expression descriptive statistics are presented in Supplementary Table 1. All effects are reported as significant at *p* < 0.05. All molecular analyses were conducted using SPSS Statistics Version 26 (IBM Corporation, 2019)**.**

## Results

### Behavioural analysis

#### Bottom dwelling tendency

Overall, time was a significant predictor of bottom dwelling tendency (Likelihood Ratio Test (LRT) = 44.9, *p* < 0.001). Pairwise comparisons of bottom-dwelling tendency at each minute, suggested that the first minute significantly differed from all other timepoints (*p* < 0.001 for all comparisons). However, the second minute did not significantly differ from subsequent timepoints in terms of bottom dwelling tendency (*p* > 0.98 for all comparisons, Fig. 1b). Since minute 2 was the earliest time point at which animals differed in behaviour, it was used to select individuals for molecular analysis (Fig. 1c,d). Zebrafish that had low bottom dwelling tendency in the second minute were classed as low stress reactive and those that maintained a high degree of bottom dwelling in minute 2 were classed as high stress reactive. When the entire 5 min period was analysed, fish that were classed as high or low stress reactive on the basis of bottom dwelling at minute 2 also grouped at the top or bottom of the population distribution for bottom dwelling over the 5 min period.

**Figure 2:**
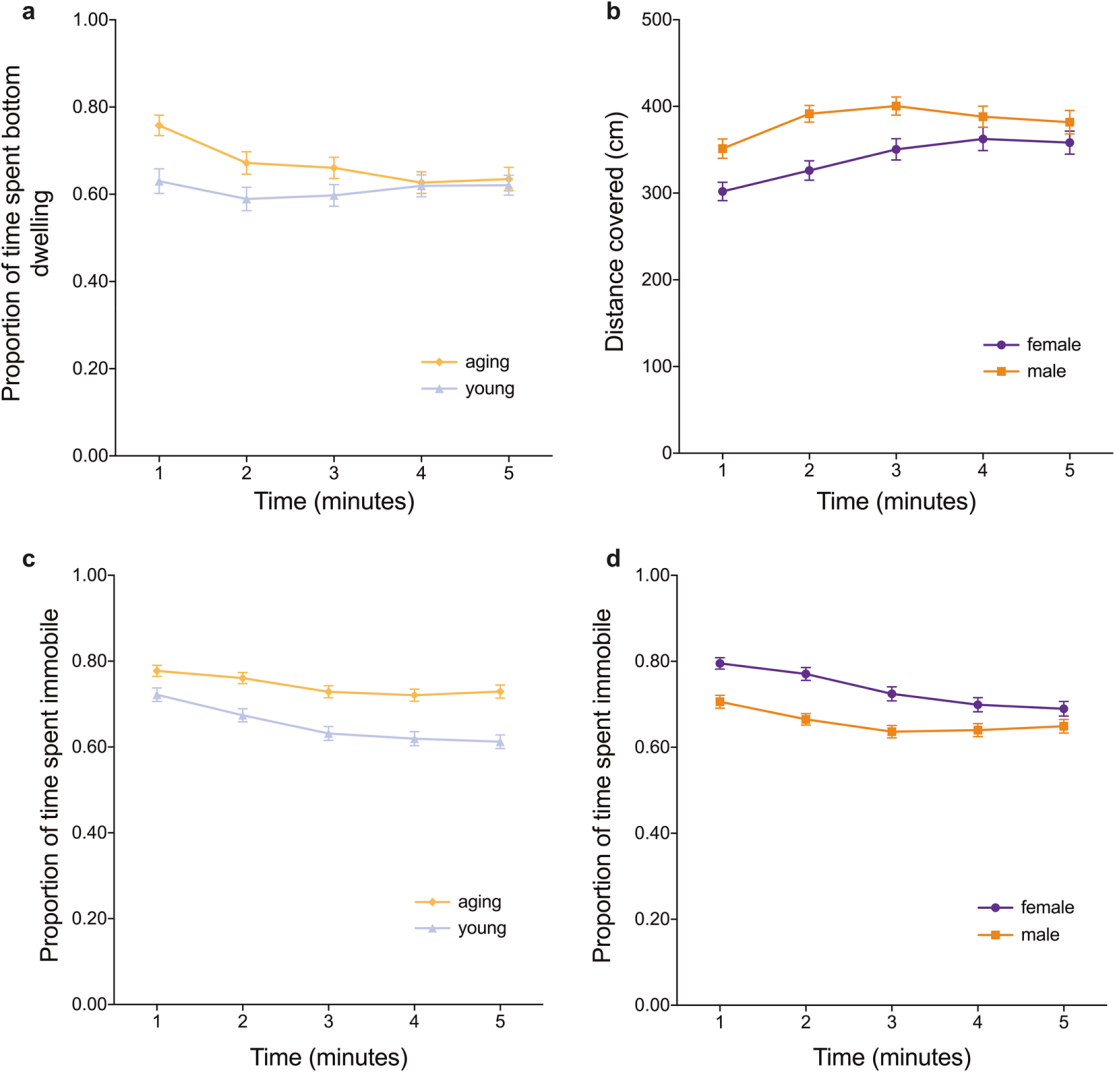
Results of the novel tank diving test. (**a**) Overall proportion of time spent in the bottom third of the tank over the duration of the test. (**b**) Distance covered by males and females for each minute of the novel tank test. (**c**) Proportion of time aging and young, and (**d**) males and females spent in an immobile state over the 5 min of the test. All error bars represent ± standard error mean (SEM).

The interaction between age and minute was a significant predictor of the proportion of time per minute spent on the bottom of the tank (LRT = 23.7, *p* < 0.001), and post-hoc analysis revealed that aging fish exhibited less bottom dwelling over time, but young fish did not change their bottom dwelling tendency over time (Fig. 2a: see Supplementary Information for additional results). The interaction between sex and minute was not significant (LRT = 1.9, *p* = 0.168), however, sex was significant as a main effect (LRT = 8.2, *p* = 0.004), with males spending more time on the bottom (*M* = 0.688, *SD* = 0.258) than females (*M* = 0.583, *SD* = 0.315;).

#### Total distance moved

The interaction between sex and minute was a significant predictor of total distance moved per minute (LRT = 7.0, *p* = 0.008), and post-hoc analysis revealed that males increased their distance moved at a faster rate than females (Fig. 2b). However, the interaction between age and minute was not significant (LRT = 2.2, *p* = 0.137), nor was age as a main effect (LRT = 2.2, *p* = 0.133).

#### Immobilization tendency

The interaction between age and minute was a significant predictor of the proportion of time per minute spent immobile (LRT = 6.5, *p* = 0.011). Aging fish spent more time immobile (proportion of time per minute spent immobile = 0.743, *SD* = 0.156) than young fish (proportion of time per minute spent immobile = 0.652, *SD* = 0.188; Fig. 2c), and post-hoc analysis revealed that aging fish resumed movement more slowly than younger fish. The interaction between sex and minute was also a significant predictor of the proportion of time per minute spent immobile (LRT = 17.8, *p* < 0.001). Females spent more time immobile (proportion of time per minute spent immobile = 0.736, *SD* = 0.177) than males (proportion of time per minute spent immobile = 0.659, *SD* = 0.173; Fig. 2d), and post-hoc analysis revealed that females resumed movement more quickly than males.

### HPI axis gene expression

**Figure 3:**
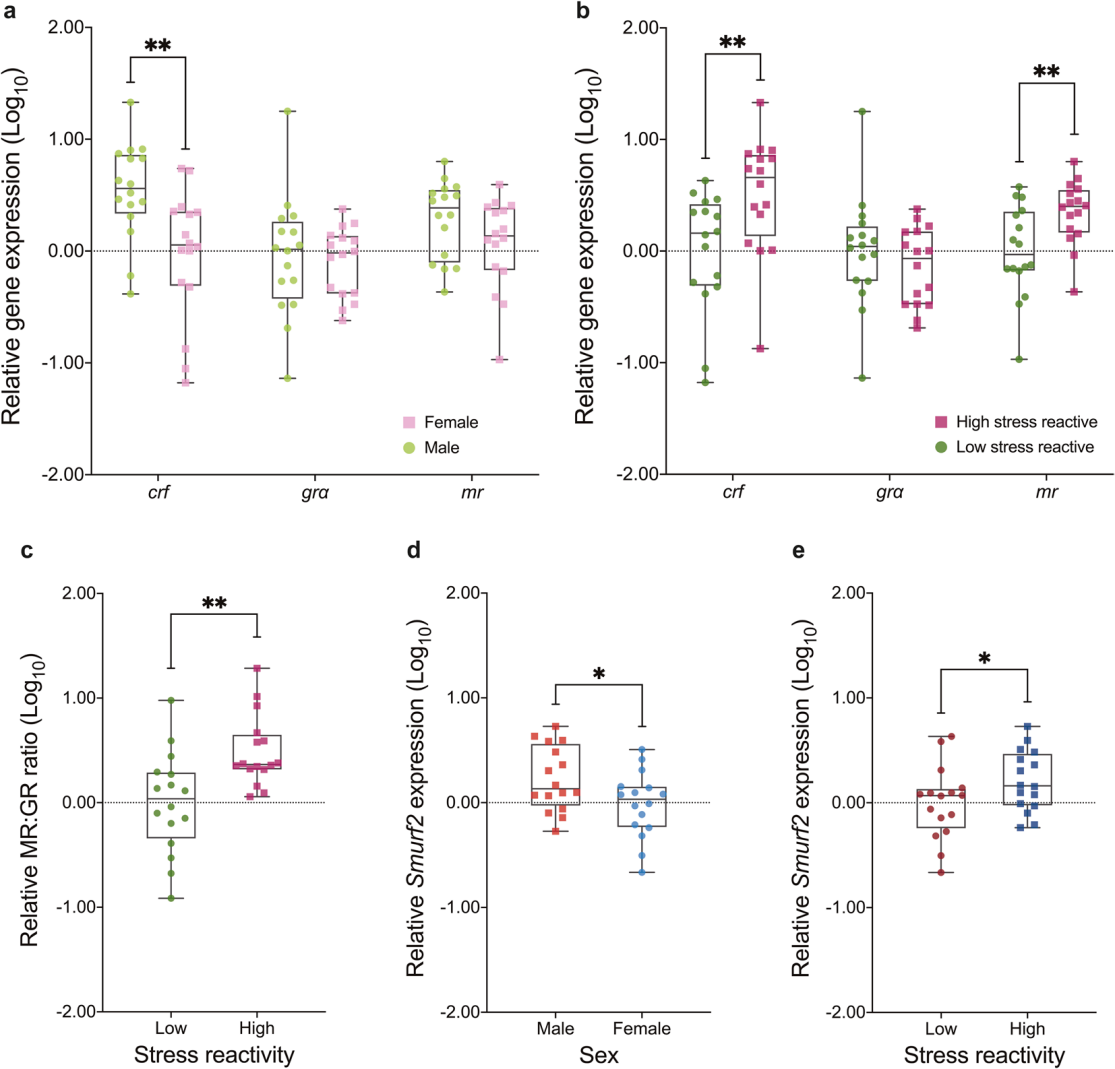
Analysis of HPI axis-associated gene expression within brain extracts by qPCR validates behaviourally determined stress reactivity. qPCR analysis of the HPI axis-associated genes, corticotropin-releasing factor (*crf*), glucocorticoid receptor α (*grα*) and mineralocorticoid receptor *(mr)* in association with zebrafish (**a**) sex and (**b**) stress reactivity (*N* = 96). (**c**) Stress reactivity had a significant effect on the MR:GR ratio, a key indicator of HPA axis dysfunction (*N* = 32). The expression of SMAD Specific E3 Ubiquitin Protein Ligase (*smurf2*) was also analysed through qPCR and was significantly associated with both (**d**) sex and (**e**) stress reactivity (*N* = 32). Box plots indicate median values (middle lines), first and third quartiles (box edges) and minimum and maximum values (whiskers). *p-value < 0.05; **p-value < 0.01.

Fish at the extremes of the tank diving distributions were assessed for differences in the expression of HPI axis genes as well as analysis of telomere length. We performed qPCR on the expression of corticotropin-releasing factor (*crf*), mineralocorticoid receptor (*mr*) and glucocorticoid receptor α (*grα*) (Fig. 3a,b). Subjects identified as more stress reactive in the novel tank assay were found to have significantly higher expression of *crf* and *mr* compared with their less stress reactive siblings; they were also found to have a significantly higher Mineralocorticoid:Glucocorticoid (MR:GR) ratio, an important factor in maintaining HPA axis homeostasis and regulating the stress response^49^ (Fig. 3c).

#### Corticotropin-releasing factor (crf)

The three-way between-subjects ANOVA revealed that the main effect of sex was significant (*F*(1,24) = 11.87, *p* = 0.002, partial η^2^ = 0.331). Males (*M* = 0.54, *SD* = 0.43) had significantly higher *crf* expression compared with females (*M* = − 0.03, *SD* = 0.58). The main effect of behavioural stress reactivity was also significant (*F*(1,24) = 9.20, *p* = 0.006, partial η^2^ = 0.277). Subjects with high stress reactivity (*M* = 0.50, *SD* = 0.52) had significantly higher *crf* expression compared with low stress reactive subjects (*M* = 0.00, *SD* = 0.54).

#### Glucocorticoid receptor alpha (grα)

The three-way between-subjects ANOVA revealed no significant main effects nor any significant interactions (Supplementary Table 3).

#### Mineralocorticoid receptor (mr)

The three-way between-subjects ANOVA revealed that the main effect of behavioural stress reactivity was significant (*F*(1,24) = 8.18, *p* = 0.009, partial η^2^ = 0.254). High stress reactive subjects (*M* = 0.35, *SD* = 0.29) had significantly higher *mr* expression compared with subjects with low stress reactivity (*M* = 0.00, *SD* = 0.41).

#### Mineralocorticoid:glucocorticoid (MR:GR) ratio

The three-way between-subjects ANOVA revealed that the main effect of behavioural stress reactivity was significant (*F*(1,24) = 11.04, *p* = 0.003, partial η^2^ = 0.315). Subjects with high behaviourally determined stress reactivity (*M* = 0.49, *SD* = 0.34) had a higher MR:GR ratio compared with low stress reactive zebrafish (*M* = 0.00, *SD* = 0.48).

### Telomere length

**Figure 4:**
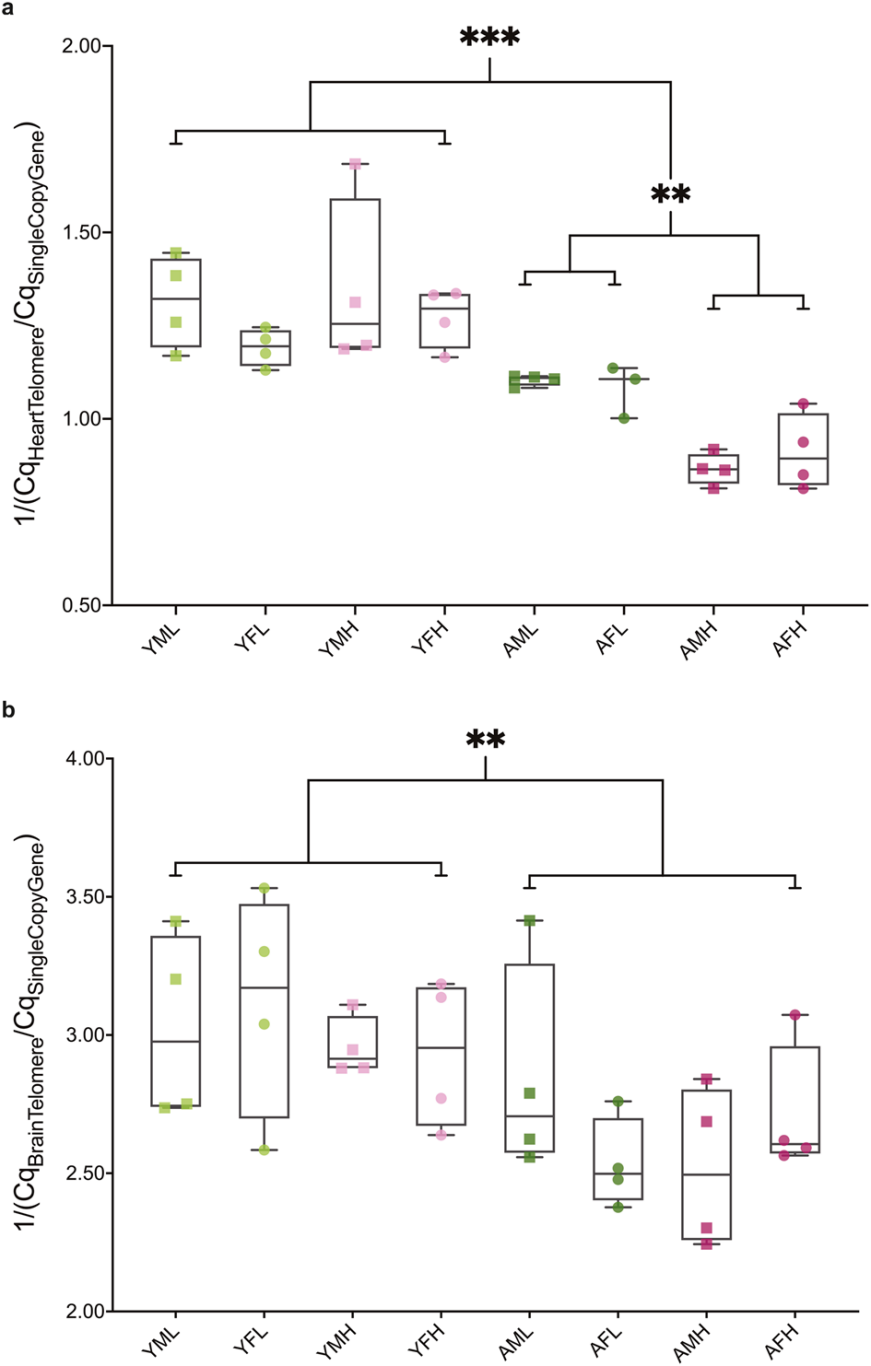
Telomere length shortens with age in both heart and brain and correlates with stress reactivity in the heart, but not brain, of zebrafish. (**a**) Relative telomere length ratios calculated by qPCR for heart tissue grouped by age, sex and stress reactivity (*N* = 31). Age and stress reactivity in aging zebrafish have a signficant effect on telomere length in heart tissue. (**b**) Relative telomere length ratios for brain grouped by age, sex and stress reactivity (*N* = 32). Only age has a significant effect. Box plots indicate median values (middle lines), first and third quartiles (box edges) and minimum and maximum values (whiskers). *YFL* young female low stress reactivity, *YFH* young female high stress reactivity, *YML* young male low stress reactivity, *YMH* young male high stress reactivity, *AFL* aging female low stress reactivity, *AFH* aging female high stress reactivity, *AML* aging male low stress reactivity, *AMH* aging male high stress reactivity. **p-value < 0.01; ***p-value < 0.001.

Telomere length was shorter in both brain and heart tissue from aging fish irrespective of the level of stress reactivity (Fig. 4a,b). Heart tissue of aging subjects with high stress reactivity had significantly shorter telomeres compared with individuals with low stress reactivity (Fig. 4a). No significant effect of stress reactivity on telomere length in brain tissue was seen. No sex differences were identified in the telomere analysis.

#### Heart Telomere Length

The three-way between-subjects ANOVA revealed that the main effect of age was significant (Fig. 4a; *F*(1,23) = 52.91, *p* < 0.001, partial η^2^ = 0.697). Shorter telomeres were found in aging subjects (*M* = 0.98, *SD* = 0.12) compared with younger subjects (*M* = 1.28, *SD* = 0.14). The interaction between age and stress reactivity was significant (*F*(1,23) = 10.71, *p* = 0.003, partial η^2^ = 0.318). The Bonferroni adjusted post hoc comparisons found that aging high stress reactive fish (*M* = 0.89, *SD* = 0.08) had significantly shorter telomeres in heart tissue compared with their low stress reactive siblings (*M* = 1.09, *SD* = 0.04) (Fig. 4a; *p* = 0.002). At both extremes of the stress reactivity spectrum, aging fish were found to have significantly shorter heart telomeres compared with their younger counterparts (young high stress reactive (*M* = 1.31, *SD* = 0.17) and young low stress reactive (*M* = 1.25, *SD* = 0.11), respectively: (*p* < 0.001; *p* = 0.011)). No other significant effects or interactions were found (Supplementary Table 3).

#### Brain telomere length

The three-way between-subjects ANOVA revealed that the main effect of age was significant (Fig. 4b: *F*(1,24) = 11.71, *p* = 0.002, partial η^2^ = 0.328). Shorter telomeres were found in aging subjects (*M* = 2.65, *SD* = 0.29) compared with younger subjects (*M* = 3.01, *SD* = 0.28). No other significant effects or interactions were found (Supplementary Table 3).

### SMAD specific E3 ubiquitin-ligase 3 (*smurf2*) expression

*smurf2* is thought to function downstream of telomere attrition to affect replicative senescence^50,51^. Therefore, we also tested whether the differences in telomere length were accompanied by differences in the expression of *smurf2* by qPCR (Fig. 3d,e). Pearson’s product-moment correlations were conducted to assess the relationship between telomere length, in both brain and heart, and *smurf2* gene expression. There was no statistically significant correlation between heart telomere length and *smurf2* expression (*r*(29) = − 0.012, *p* = 0.951), nor was there a statistically significant correlation between brain telomere length and *smurf2* expression (*r*(30) = 0.173, *p* = 0.345).

Nonetheless, the three-way between-subjects ANOVA revealed that the main effect of sex was significant (*F*(1,24) = 6.16, *p* = 0.020, partial η^2^ = 0.204). Males (*M* = 0.18, *SD* = 0.34) had significantly higher *smurf2* expression compared with females (*M* = − 0.02, *SD* = 0.43). The main effect of behavioural stress reactivity was also significant (*F*(1,24) = 4.38, *p* = 0.047, partial η^2^ = 0.154). Zebrafish with high stress reactivity (*M* = 0.21, *SD* = 0.29) had significantly higher *smurf2* expression compared with fish with low stress reactivity (*M* = 0.00, *SD* = 0.35). The two-way interaction between age and behavioural stress reactivity was significant (*F*(1,24) = 4.67, *p* = 0.041, partial η^2^ = 0.163). The Bonferroni adjusted post hoc comparisons found that aging high stress reactive fish (*M* = 0.29, *SD* = 0.23) had significantly higher *smurf2* expression compared with aging zebrafish with low stress reactivity (*M* = − 0.13, *SD* = 0.41, *p* = 0.006). The three-way interaction between age, sex and behavioural stress reactivity was also significant (*F*(1,24) = 4.46, *p* = 0.045, partial η^2^ = 0.157). The Bonferroni adjusted post hoc pairwise comparisons revealed that aging female subjects with high stress reactivity (*M* = 0.29, *SD* = 0.21) had significantly higher *smurf2* expression compared with aging female fish with low stress reactivity (*M* = − 0.34, *SD* = 0.35, *p* = 0.004). Post hoc comparisons also revealed that aging high stress reactive female zebrafish had significantly higher *smurf2* expression compared with younger high stress reactive female fish (*M* = − 0.12, *SD* = 0.12, *p* = 0.048) whilst aging low stress reactive female zebrafish were found to have significantly lower *smurf2* expression compared with younger females with low stress reactivity (*M* = 0.09, *SD* = 0.17, *p* = 0.039). Additionally, aging low stress reactive male zebrafish (*M* = 0.08, *SD* = 0.40) demonstrated significantly higher *smurf2* expression compared with aging low stress reactive female fish (*p* = 0.046). Finally, younger high stress reactive male zebrafish (*M* = 0.37, *SD* = 0.29) exhibited significantly higher *smurf2* expression compared with younger high stress reactive female fish (*p* = 0.020).

Pearson’s product-moment correlations were performed to assess the relationship between HPI axis genes, including the MR:GR ratio, and *smurf2* expression. The correlation between *crf* and *smurf2* was significant (*r*(30) = 0.487, *p* = 0.005), with *crf* expression accounting for 24% of the variance in *smurf2* expression. No other correlations were statistically significant (*p* > 0.05).

## Discussion

The present study used zebrafish to test the hypothesis that stress reactivity exacerbates age-dependent telomere shortening in tissues relevant to age-related disease. Importantly, our findings suggest that high stress reactivity may contribute to an increased rate of telomere attrition in a tissue-specific manner, with stress reactivity correlating with telomere length in the heart, but not brain, of zebrafish. Furthermore, our study demonstrates the value of zebrafish as a translational model to explore associations between stress reactivity, cellular aging, and age-related disease.

Zebrafish have been shown to demonstrate persistent individual differences in behaviour across both time and context and, as seen in humans^52,53^ and rodents^54–56^, show increased anxiety-like behaviour with age^57^. Thus, zebrafish provide a promising animal model for studying associations between behaviours associated with specific personality traits and aging^41^. Our finding that aging zebrafish exhibited increased anxiety-like behaviours is in line with previous findings. In agreement with others^58^, we also observed sexually dimorphic behavioural responses in both young and aging zebrafish; males showed increased bottom-dwelling, covered longer distances, and were more mobile than females when exposed to a novel environment. Importantly, males also had significantly higher *crf* expression, a key regulator of locomotor activity^59^, thus reflecting consistency between behavioural and molecular data. In contrast to other research^60^, no sex differences in telomere length were observed. Given that both behavioural stress reactivity and HPI activity were found to be sexually dimorphic, the lack of sex differences in telomere length may indicate that females are more sensitive to the effect of HPI activity on telomere length. Alternatively, it may be that the subtle differences in HPI activity identified between the sexes were insufficient to elicit a significant effect for sex on telomere length with the sample size used here. However, as we did not directly test the impact of HPI activity on telomere length in male and female fish, further experiments are necessary to test these hypotheses.

Cardiovascular diseases have been widely associated with aging^61^, anxious disposition^62^, and telomere shortening^63^. The data reported here support such an association and points to a possible mechanism: zebrafish with high stress reactivity, and therefore a likely increased lifetime of perceived stress, have an increased HPI axis response which, over their lifetime, may lead to an increased rate of telomere shortening in heart tissue. Importantly, increased levels of stress hormone, such as cortisol and glucocorticoids, have been previously reported to have a negative effect on telomere length in peripheral tissues^63–65^. Furthermore, the expression of telomerase, the enzyme with the ability to counteract telomere erosion, is susceptible to modulation by different hormones^39,65–67^, including glucocorticoids^68^, thus providing further support for such a molecular mechanism. Interestingly, the balance of cardiomyocyte *mr* and *gr* has been shown to be paramount for cardiovascular health in mice^69^, which is consistent with the higher MR:GR ratio found here in aging zebrafish with shorter telomeres.

Although zebrafish retain telomerase activity during their adult life^38,40^, our data shows a significant decline in telomere length with age, which may be exacerbated by HPI axis dysfunction. In most vertebrates, including zebrafish, the major source of stem cells in the heart are the resident cardiomyocytes, which are capable of temporarily increasing their telomerase activity in response to injury^40,70–72^. However, when this peak is weakened by stress-related hormones^26,39,63^, it may diminish their ability to counteract cardiac damage, thus leading to shorter telomeres and the associated increased risk of cardiomyopathies. It should also be noted that our research cannot rule out a telomerase-independent process resulting from a chronically activated HPI axis, such as the postulated direct effect of reactive oxygen species on telomere length^73^. Nonetheless, the present investigation demonstrates that zebrafish are a promising animal model in which to study the association between stress reactivity and cardiomyopathies in aging individuals.

Upregulation of the ubiquitin ligase enzyme *smurf2* is suggested to be a consequence of telomere attrition in human fibroblasts, and to be sufficient to drive senescence in the absence of DNA damage or detectable cellular stress^50,51^. However, telomere shortening did not predict *smurf2* expression in our study. Instead, we found that *smurf2* expression was increased in the anxious phenotype regardless of age or telomere length. HPA activity has been previously linked with the upregulation of ubiquitin ligases. For example, the expression of *smurf2*, among other ligases, is associated with social stress in the ventromedial prefrontal cortex of monkeys^74^, and glucocorticoid receptor imbalance is associated with the ubiquitin ligase Pellino-1^75^. Here, we find an association between *crf* and the ubiquitin ligase *smurf2*. Thus, our findings suggest that the increase of *smurf2* is not a consequence of telomere shortening, but rather of increased HPI activity. In accordance with others^50^, we also observed a sexual dimorphic pattern with increased expression of *smurf2* in males.

A growing body of literature links shorter telomeres from peripheral tissues, such as leukocytes, with the onset and progression of neurodegenerative and psychiatric disorders^28–30,76^. Importantly, perceived stress, or stress-reactivity, is hypothesised to be a mediating factor in this relationship^25,66,76,77^. However, the present study finds no effect for stress reactivity on the telomere length of brain tissue. This disparity of effect on heart and brain telomere length suggests that stress reactivity influences telomere length in a tissue-specific manner. Thus, the assessment of leukocyte telomere length, or other proxy tissues, may not faithfully reflect telomere dynamics in the brain. It is therefore important to not overinterpret telomere length results obtained from peripheral tissues in clinical studies. Our use of a zebrafish model here has overcome this hurdle, enabling human-like telomere length^39,40^ to be assessed directly in disease-relevant tissues.

Although we demonstrate a clear association between stress reactivity and telomere length in heart tissue and show that stress reactivity affects telomere length in a tissue specific manner, there are limitations to our study. Differential rates of telomere shortening within different brain regions in humans have been previously described, with telomere length in the hippocampus significantly different between individuals with depression and controls^67,78^. Similarly, differences in the rate of telomere erosion have been observed for different cell types^79,80^. Therefore, it is possible that tissue heterogeneity could be masking anxiety-driven differences within zebrafish whole brain extracts in our study, a consideration that requires further investigation. It is also worth noting that whilst we report telomere shortening at 18 months of age in both brain and heart tissue, previous research using zebrafish has not observed age-dependent differences in telomere length until fish are at least 2 years of age^39,81^, with other research finding no telomere length effect of age in the zebrafish brain^36^. This discrepancy may highlight possible experimental differences between studies, namely the technique of telomere length analysis or strain differences^38,39^, or may reflect a lower rate of cell renewal and neurogenesis in the adult brain compared to heart^36,73,80^, making detection of shortening challenging. Therefore, if stress reactivity does lead to exacerbated telomere shortening in the brain, it may only be possible to observe this at much older stages in the zebrafish lifespan.

Taken together, we have demonstrated, using a population-based zebrafish strategy, that elevated stress reactivity affects telomere length in a tissue-specific manner, exacerbating age-dependent telomere shortening in heart but not brain tissue. As such, we demonstrate the importance of assessing telomere length in disease-relevant tissues to obtain meaningful results, and highlight the possible importance of both stress reactivity, and the resulting exacerbated telomere shortening, in the onset and progression of cardiovascular diseases. Finally, we demonstrate the utility and importance of zebrafish as a translational model that can be used in future studies to assess associations between stress reactivity, cellular aging and age-related disease.

## Supplementary Information

Supplementary Information
