# Supplementary material for: Stress reactivity elicits a tissue-specific reduction in telomere length in aging zebrafish (Danio rerio)
Source: Sci Rep. 2021 Jan 11;11:339. doi: 10.1038/s41598-020-79615-1 (PMC7801459; doi:10.1038/s41598-020-79615-1)
Supplement: Supplementary file 1 — Supplementary Information [file 41598_2020_79615_MOESM1_ESM.doc]

Stress reactivity elicits a tissue-specific reduction in telomere length in aging zebrafish (*Danio rerio*)

**Author line:** James R. Evans^1+^, Jose V. Torres-Pérez^1+^, Maria Elena Miletto Petrazzini^1,2^, Riva Riley^1^, Caroline H. Brennan^1^*

**Author affiliation:** ^1^School of Biological and Chemical Sciences, Queen Mary University of London, Mile End Rd, E1 4NS, UK.

^2^Department of Biomedical Sciences, University of Padova, Via Ugo Bassi 58/B, 35131, Padova, Italy

^+^Equal contribution

**Corresponding author:** [*c.h.brennan@qmul.ac.uk](about:blank)

**SUPPLEMENTARY MATERIAL**

**Supplementary information for behavioural statistical methodology:**

All statistical analyses were carried out in R version 3.2.2 (R core developer team), and linear mixed effects models (LME) were fitted using the lme4 package (Bates et al., 2015); generalized mixed effect models (GLMM) were fitted using the glmmTMB package (Brooks et al., 2017). Data distributions were initially assessed visually, and model diagnostics were subsequently checked to assure appropriate fits. For all models, predictors were tested as main effects when interaction terms were not signficant. We hence fitted LMEs for normally distributed data (total distance moved) and GLMMs with betabinomial error distributions where binomial models had been over-dispersed (Bolker et al., 2009).

To choose a timepoint at which to select individuals for our molecular analysis of telomere length, we performed a Tukey posthoc test on the bottom dwelling data pooled across age and sex. We constructed a betabinomial GLMM based on this pooled data with the proportion of time spent swimming on the bottom as the response variable, minute as the fixed effect, and batch number and individual ID as random effects. We used the emmeans package in R (Lenth, 2019) to perform the Tukey posthoc and analyze which timepoint represented the earliest ‘recovery’ from the stress.

To assess the effect of age and sex on the tendency to swim on the bottom over the course of the experiment, we ran a betabinomial GLMM with the proportion of time spent swimming on the bottom in each minute as the response variable, the interaction between age and minute and the interaction between sex and minute as fixed effects, and individual ID and batch as random effects. Interactions that were not significant were subsequently refitted as main effects.

To assess the effect of age and sex on the tendency to remain immobile over the course of the experiment, we ran a betabinomial GLMM with the proportion of time spent swimming on the bottom in each minute as the response variable, the interaction between age and minute and the interaction between sex and minute as fixed effects, and individual ID and batch as random effects.

**Supplementary Information for additional behavioural results:**

Bottom dwelling tendency

A post-hoc analysis revealed that aging fish exhibited a significant decrease in bottom dwelling tendency from minute one to minutes two, three, four, and five (*p* < 0.001), while young fish did not show a change in bottom dwelling tendency from minute one to any other minute (*p* = 0.247; Fig. 2a). No other comparisons between minutes two, three, four, or five were significant in either age group.

Total distance moved

A post-hoc analysis revealed that males exhibited a significant increase in distance moved from minute one to minute two (*p* = 0.005), while females did not show a significant increase in distance moved from minute one to minute two (*p* = 0.452); rather, females exhibited a significant increase in distance moved from minute one to minute three (*p* < 0.001) and all other minutes (*p* < 0.001, Fig. 2b).

Immobilization tendency

A post-hoc analysis revealed that aging fish did not exhibit a change in immobilization from minute one to minute two (*p* = 0.938) but did exhibit a significant decrease in immobilization from minute one to all subsequent minutes (*p* < 0.001); no other comparisons were significant. Younger fish exhibited significantly decreased immobilization from minute one to minute two *(p* = 0.003) as well as between minutes one and two and all subsequent minutes (p < 0.040, Fig. 2c). In addition, a post-hoc test revealed that females first exhibited a significantly lower immobilization tendency between minute one and minute two (*p* = 0.003), whereas males did not exhibit a significant different between minute one and minute two (*p* = 0.938); rather, their immobilization tendencies was first significantly different between minute one and minute three (*p* = 0.001, Fig. 2d).

**Supplementary Table 1. Descriptive statistics for molecular data**

| **Main effects:** | | **Age** | | | | |  | **Sex** | | | | |  | **Stress reactivity** | | | | |
| --- | --- | --- | --- | --- | --- | --- | --- | --- | --- | --- | --- | --- | --- | --- | --- | --- | --- | --- |
|  |  | **Aging** | |  | **Young** | |  | **Male** | |  | **Female** | |  | **High** | |  | **Low** | |
|  |  | ***M*** | ***SD*** |  | ***M*** | ***SD*** |  | ***M*** | ***SD*** |  | ***M*** | ***SD*** |  | ***M*** | ***SD*** |  | ***M*** | ***SD*** |
| Heart Telomere Length | | 0.98 | 0.12 |  | 1.28 | 0.14 |  | 1.16 | 0.23 |  | 1.12 | 0.16 |  | 1.10 | 0.25 |  | 1.18 | 0.12 |
| Brain Telomere Length | | 2.65 | 0.29 |  | 3.01 | 0.28 |  | 2.84 | 0.34 |  | 2.82 | 0.34 |  | 2.78 | 0.28 |  | 2.88 | 0.38 |
| Corticotropin-releasing factor | | 3.93 | 5.08 |  | 2.92 | 2.87 |  | 5.18 | 5.03 |  | 1.68 | 1.64 |  | 5.23 | 5.10 |  | 1.62 | 1.26 |
| Glucocorticoid receptor alpha | | 1.86 | 4.32 |  | 1.22 | 0.51 |  | 2.08 | 4.25 |  | 0.99 | 0.62 |  | 0.95 | 0.68 |  | 2.13 | 4.23 |
| Mineralocorticoid receptor | | 1.86 | 1.56 |  | 2.19 | 1.32 |  | 2.48 | 1.64 |  | 1.57 | 1.05 |  | 2.63 | 1.48 |  | 1.42 | 1.12 |
| Mineralocorticoid:  Glucocorticoid Ratio | | 4.17 | 5.22 |  | 1.96 | 1.19 |  | 3.70 | 4.88 |  | 2.43 | 2.57 |  | 4.37 | 4.73 |  | 1.77 | 2.30 |
| SMAD specific E3 ubiquitin-ligase 3 | | 1.66 | 1.28 |  | 1.66 | 1.32 |  | 2.12 | 1.50 |  | 1.20 | 0.82 |  | 1.98 | 1.35 |  | 1.34 | 1.16 |
| **Two-way interactions:** | | **Age and sex** | | | | | | | | | | |  |  |  |  |  |  |
|  |  | **Aging** | | | | |  | **Young** | | | | |  |  |  |  |  |  |
|  |  | **Male** | |  | **Female** | |  | **Male** | |  | **Female** | |  |  |  |  |  |  |
|  |  | ***M*** | ***SD*** |  | ***M*** | ***SD*** |  | ***M*** | ***SD*** |  | ***M*** | ***SD*** |  |  |  |  |  |  |
| Heart Telomere Length | | 0.98 | 0.13 |  | 0.98 | 0.12 |  | 1.33 | 0.17 |  | 1.23 | 0.08 |  |  |  |  |  |  |
| Brain Telomere Length | | 2.68 | 0.36 |  | 2.62 | 0.21 |  | 2.99 | 0.23 |  | 3.02 | 0.33 |  |  |  |  |  |  |
| Corticotropin-releasing factor | | 5.90 | 6.64 |  | 1.97 | 1.54 |  | 4.46 | 3.01 |  | 1.39 | 1.79 |  |  |  |  |  |  |
| Glucocorticoid receptor alpha | | 2.82 | 6.11 |  | 0.89 | 0.74 |  | 1.34 | 0.51 |  | 1.09 | 0.50 |  |  |  |  |  |  |
| Mineralocorticoid receptor | | 2.16 | 2.05 |  | 1.56 | 0.91 |  | 2.80 | 1.15 |  | 1.58 | 1.24 |  |  |  |  |  |  |
| Mineralocorticoid:  Glucocorticoid Ratio | | 5.34 | 6.68 |  | 3.00 | 3.25 |  | 2.06 | 0.48 |  | 1.87 | 1.67 |  |  |  |  |  |  |
| SMAD specific E3 ubiquitin-ligase 3 | | 1.98 | 1.46 |  | 1.35 | 1.08 |  | 2.27 | 1.62 |  | 1.05 | 0.47 |  |  |  |  |  |  |
|  |  | **Age and stress reactivity** | | | | | | | | | | |  |  |  |  |  |  |
|  |  | **Aging** | | | | |  | **Young** | | | | |  |  |  |  |  |  |
|  |  | **High** | |  | **Low** | |  | **High** | |  | **Low** | |  |  |  |  |  |  |
|  |  | ***M*** | ***SD*** |  | ***M*** | ***SD*** |  | ***M*** | ***SD*** |  | ***M*** | ***SD*** |  |  |  |  |  |  |
| Heart Telomere Length | | 0.89 | 0.08 |  | 1.09 | 0.04 |  | 1.31 | 0.17 |  | 1.25 | 0.11 |  |  |  |  |  |  |
| Brain Telomere Length | | 2.62 | 0.27 |  | 2.69 | 0.32 |  | 2.94 | 0.19 |  | 3.07 | 0.35 |  |  |  |  |  |  |
| Corticotropin-releasing factor | | 6.21 | 6.49 |  | 1.65 | 1.11 |  | 4.25 | 3.39 |  | 1.60 | 1.47 |  |  |  |  |  |  |
| Glucocorticoid receptor alpha | | 0.71 | 0.74 |  | 3.00 | 6.03 |  | 1.19 | 0.56 |  | 1.25 | 0.48 |  |  |  |  |  |  |
| Mineralocorticoid receptor | | 2.44 | 1.81 |  | 1.28 | 1.08 |  | 2.82 | 1.14 |  | 1.56 | 1.22 |  |  |  |  |  |  |
| Mineralocorticoid:  Glucocorticoid Ratio | | 6.08 | 6.33 |  | 2.26 | 3.14 |  | 2.65 | 1.04 |  | 1.28 | 0.94 |  |  |  |  |  |  |
| SMAD specific E3 ubiquitin-ligase 3 | | 2.18 | 1.05 |  | 1.15 | 1.34 |  | 1.77 | 1.64 |  | 1.54 | 1.00 |  |  |  |  |  |  |
|  |  | **Sex and stress reactivity** | | | | | | | | | | |  |  |  |  |  |  |
|  |  | **Male** | | | | |  | **Female** | | | | |  |  |  |  |  |  |
|  |  | **High** | |  | **Low** | |  | **High** | |  | **Low** | |  |  |  |  |  |  |
|  |  | ***M*** | ***SD*** |  | ***M*** | ***SD*** |  | ***M*** | ***SD*** |  | ***M*** | ***SD*** |  |  |  |  |  |  |
| Heart Telomere Length | | 1.11 | 0.30 |  | 1.21 | 0.14 |  | 1.09 | 0.21 |  | 1.14 | 0.08 |  |  |  |  |  |  |
| Brain Telomere Length | | 2.74 | 0.31 |  | 2.94 | 0.35 |  | 2.82 | 0.26 |  | 2.82 | 0.42 |  |  |  |  |  |  |
| Corticotropin-releasing factor | | 8.13 | 5.71 |  | 2.23 | 1.34 |  | 2.33 | 2.00 |  | 1.02 | 0.88 |  |  |  |  |  |  |
| Glucocorticoid receptor alpha | | 0.94 | 0.65 |  | 3.22 | 5.95 |  | 0.96 | 0.76 |  | 1.03 | 0.50 |  |  |  |  |  |  |
| Mineralocorticoid receptor | | 2.99 | 1.91 |  | 1.97 | 1.23 |  | 2.27 | 0.86 |  | 0.87 | 0.70 |  |  |  |  |  |  |
| Mineralocorticoid:  Glucocorticoid Ratio | | 5.00 | 6.19 |  | 2.40 | 2.97 |  | 3.73 | 2.94 |  | 1.13 | 1.24 |  |  |  |  |  |  |
| SMAD specific E3 ubiquitin-ligase 3 | | 2.51 | 1.52 |  | 1.72 | 1.47 |  | 1.44 | 0.96 |  | 0.96 | 0.63 |  |  |  |  |  |  |
| **Three-way interaction:** | | **Age, sex and stress reactivity** | | | | | | | | | | |  |  |  |  |  |  |
|  |  | **Ageing** | | | | | | | | | | |  |  |  |  |  |  |
|  |  | **Male** | | | | |  | **Female** | | | | |  |  |  |  |  |  |
|  |  | **High** | |  | **Low** | |  | **High** | |  | **Low** | |  |  |  |  |  |  |
|  |  | ***M*** | ***SD*** |  | ***M*** | ***SD*** |  | ***M*** | ***SD*** |  | ***M*** | ***SD*** |  |  |  |  |  |  |
| Heart Telomere Length | | 0.87 | 0.04 |  | 1.10 | 0.01 |  | 0.91 | 0.10 |  | 1.08 | 0.07 |  |  |  |  |  |  |
| Brain Telomere Length | | 2.52 | 0.29 |  | 2.85 | 0.39 |  | 2.71 | 0.24 |  | 2.53 | 0.16 |  |  |  |  |  |  |
| Corticotropin-releasing factor | | 9.71 | 7.90 |  | 2.08 | 1.26 |  | 2.72 | 1.79 |  | 1.21 | 0.90 |  |  |  |  |  |  |
| Glucocorticoid receptor alpha | | 0.40 | 0.23 |  | 5.25 | 8.44 |  | 1.02 | 0.99 |  | 0.76 | 0.50 |  |  |  |  |  |  |
| Mineralocorticoid receptor | | 2.62 | 2.67 |  | 1.70 | 1.43 |  | 2.26 | 0.65 |  | 0.85 | 0.43 |  |  |  |  |  |  |
| Mineralocorticoid:  Glucocorticoid Ratio | | 7.76 | 8.31 |  | 2.93 | 4.41 |  | 4.41 | 4.12 |  | 1.58 | 1.55 |  |  |  |  |  |  |
| SMAD specific E3 ubiquitin-ligase 3 | | 2.26 | 1.29 |  | 1.70 | 1.76 |  | 2.10 | 0.96 |  | 0.60 | 0.54 |  |  |  |  |  |  |
|  |  | **Young** | | | | | | | | | | |  |  |  |  |  |  |
|  |  | **Male** | | | | |  | **Female** | | | | |  |  |  |  |  |  |
|  |  | **High** | |  | **Low** | |  | **High** | |  | **Low** | |  |  |  |  |  |  |
|  |  | ***M*** | ***SD*** |  | ***M*** | ***SD*** |  | ***M*** | ***SD*** |  | ***M*** | ***SD*** |  |  |  |  |  |  |
| Heart Telomere Length | | 1.35 | 0.23 |  | 1.31 | 0.12 |  | 1.27 | 0.08 |  | 1.19 | 0.05 |  |  |  |  |  |  |
| Brain Telomere Length | | 2.95 | 0.11 |  | 3.03 | 0.34 |  | 2.93 | 0.27 |  | 3.11 | 0.41 |  |  |  |  |  |  |
| Corticotropin-releasing factor | | 6.54 | 2.65 |  | 2.37 | 1.60 |  | 1.95 | 2.40 |  | 0.82 | 0.96 |  |  |  |  |  |  |
| Glucocorticoid receptor alpha | | 1.49 | 0.39 |  | 1.20 | 0.63 |  | 0.89 | 0.58 |  | 1.30 | 0.37 |  |  |  |  |  |  |
| Mineralocorticoid receptor | | 3.36 | 0.98 |  | 2.24 | 1.13 |  | 2.29 | 1.15 |  | 0.88 | 0.98 |  |  |  |  |  |  |
| Mineralocorticoid:  Glucocorticoid Ratio | | 2.24 | 0.13 |  | 1.88 | 0.66 |  | 3.06 | 1.44 |  | 0.67 | 0.80 |  |  |  |  |  |  |
| SMAD specific E3 ubiquitin-ligase 3 | | 2.77 | 1.88 |  | 1.76 | 1.39 |  | 0.78 | 0.21 |  | 1.32 | 0.53 |  |  |  |  |  |  |

**Supplementary Table 2. Descriptive statistics (log 10)** for gene expression data

| **Main effects:** | | **Age** | | | | |  | **Sex** | | | | |  | **Stress reactivity** | | | | |
| --- | --- | --- | --- | --- | --- | --- | --- | --- | --- | --- | --- | --- | --- | --- | --- | --- | --- | --- |
|  |  | **Aging** | |  | **Young** | |  | **Male** | |  | **Female** | |  | **High** | |  | **Low** | |
|  |  | ***M*** | ***SD*** |  | ***M*** | ***SD*** |  | ***M*** | ***SD*** |  | ***M*** | ***SD*** |  | ***M*** | ***SD*** |  | ***M*** | ***SD*** |
| Corticotropin-releasing factor | | 0.34 | 0.53 |  | 0.16 | 0.63 |  | 0.54 | 0.43 |  | -0.03 | 0.58 |  | 0.50 | 0.52 |  | 0.00 | 0.54 |
| Glucocorticoid receptor alpha | | -0.19 | 0.56 |  | 0.04 | 0.21 |  | -0.05 | 0.54 |  | -0.10 | 0.31 |  | -0.14 | 0.35 |  | 0.00 | 0.50 |
| Mineralocorticoid receptor | | 0.14 | 0.32 |  | 0.21 | 0.43 |  | 0.28 | 0.35 |  | 0.06 | 0.41 |  | 0.35 | 0.29 |  | 0.00 | 0.41 |
| Mineralocorticoid:  Glucocorticoid Ratio | | 0.32 | 0.54 |  | 0.16 | 0.42 |  | 0.33 | 0.46 |  | 0.16 | 0.50 |  | 0.49 | 0.34 |  | 0.00 | 0.48 |
| SMAD specific E3 ubiquitin-ligase 3 | | 0.08 | 0.39 |  | 0.13 | 0.27 |  | 0.22 | 0.31 |  | -0.02 | 0.32 |  | 0.21 | 0.29 |  | 0.00 | 0.35 |
| **Two-way interactions:** | | **Age and sex** | | | | | | | | | | |  |  |  |  |  |  |
|  |  | **Aging** | | | | |  | **Young** | | | | |  |  |  |  |  |  |
|  |  | **Male** | |  | **Female** | |  | **Male** | |  | **Female** | |  |  |  |  |  |  |
|  |  | ***M*** | ***SD*** |  | ***M*** | ***SD*** |  | ***M*** | ***SD*** |  | ***M*** | ***SD*** |  |  |  |  |  |  |
| Corticotropin-releasing factor | | 0.57 | 0.46 |  | 0.12 | 0.52 |  | 0.51 | 0.43 |  | -0.19 | 0.62 |  |  |  |  |  |  |
| Glucocorticoid receptor alpha | | -0.19 | 0.73 |  | -0.18 | 0.36 |  | 0.10 | 0.19 |  | -0.01 | 0.24 |  |  |  |  |  |  |
| Mineralocorticoid receptor | | 0.17 | 0.41 |  | 0.11 | 0.32 |  | 0.40 | 0.25 |  | 0.02 | 0.51 |  |  |  |  |  |  |
| Mineralocorticoid:  Glucocorticoid Ratio | | 0.36 | 0.66 |  | 0.29 | 0.42 |  | 0.30 | 0.11 |  | 0.03 | 0.57 |  |  |  |  |  |  |
| SMAD specific E3 ubiquitin-ligase 3 | | 0.18 | 0.34 |  | -0.02 | 0.43 |  | 0.27 | 0.29 |  | -0.01 | 0.18 |  |  |  |  |  |  |
|  |  | **Age and stress reactivity** | | | | | | | | | | |  |  |  |  |  |  |
|  |  | **Aging** | | | | |  | **Young** | | | | |  |  |  |  |  |  |
|  |  | **High** | |  | **Low** | |  | **High** | |  | **Low** | |  |  |  |  |  |  |
|  |  | ***M*** | ***SD*** |  | ***M*** | ***SD*** |  | ***M*** | ***SD*** |  | ***M*** | ***SD*** |  |  |  |  |  |  |
| Corticotropin-releasing factor | | 0.63 | 0.49 |  | 0.05 | 0.51 |  | 0.38 | 0.63 |  | -0.05 | 0.60 |  |  |  |  |  |  |
| Glucocorticoid receptor alpha | | -0.31 | 0.37 |  | -0.07 | 0.71 |  | 0.02 | 0.25 |  | 0.07 | 0.18 |  |  |  |  |  |  |
| Mineralocorticoid receptor | | 0.27 | 0.36 |  | 0.00 | 0.31 |  | 0.42 | 0.19 |  | 0.00 | 0.52 |  |  |  |  |  |  |
| Mineralocorticoid:  Glucocorticoid Ratio | | 0.58 | 0.45 |  | 0.07 | 0.51 |  | 0.40 | 0.16 |  | -0.07 | 0.48 |  |  |  |  |  |  |
| SMAD specific E3 ubiquitin-ligase 3 | | 0.29 | 0.23 |  | -0.13 | 0.41 |  | 0.12 | 0.33 |  | 0.13 | 0.22 |  |  |  |  |  |  |
|  |  | **Sex and stress reactivity** | | | | | | | | | | |  |  |  |  |  |  |
|  |  | **Male** | | | | |  | **Female** | | | | |  |  |  |  |  |  |
|  |  | **High** | |  | **Low** | |  | **High** | |  | **Low** | |  |  |  |  |  |  |
|  |  | ***M*** | ***SD*** |  | ***M*** | ***SD*** |  | ***M*** | ***SD*** |  | ***M*** | ***SD*** |  |  |  |  |  |  |
| Corticotropin-releasing factor | | 0.84 | 0.26 |  | 0.24 | 0.36 |  | 0.17 | 0.51 |  | -0.24 | 0.59 |  |  |  |  |  |  |
| Glucocorticoid receptor alpha | | -0.14 | 0.37 |  | 0.05 | 0.68 |  | -0.14 | 0.36 |  | -0.05 | 0.27 |  |  |  |  |  |  |
| Mineralocorticoid receptor | | 0.36 | 0.38 |  | 0.21 | 0.31 |  | 0.33 | 0.16 |  | -0.21 | 0.41 |  |  |  |  |  |  |
| Mineralocorticoid:  Glucocorticoid Ratio | | 0.50 | 0.39 |  | 0.16 | 0.48 |  | 0.47 | 0.31 |  | -0.16 | 0.47 |  |  |  |  |  |  |
| SMAD specific E3 ubiquitin-ligase 3 | | 0.33 | 0.27 |  | 0.12 | 0.33 |  | 0.08 | 0.27 |  | -0.02 | 0.32 |  |  |  |  |  |  |
| **Three-way interaction:** | | **Age, sex and stress reactivity** | | | | | | | | | | |  |  |  |  |  |  |
|  |  | **Ageing** | | | | | | | | | | |  |  |  |  |  |  |
|  |  | **Male** | | | | |  | **Female** | | | | |  |  |  |  |  |  |
|  |  | **High** | |  | **Low** | |  | **High** | |  | **Low** | |  |  |  |  |  |  |
|  |  | ***M*** | ***SD*** |  | ***M*** | ***SD*** |  | ***M*** | ***SD*** |  | ***M*** | ***SD*** |  |  |  |  |  |  |
| Corticotropin-releasing factor | | 0.90 | 0.31 |  | 0.23 | 0.34 |  | 0.36 | 0.29 |  | -0.13 | 0.63 |  |  |  |  |  |  |
| Glucocorticoid receptor alpha | | -0.44 | 0.23 |  | 0.07 | 1.01 |  | -0.17 | 0.46 |  | -0.20 | 0.31 |  |  |  |  |  |  |
| Mineralocorticoid receptor | | 0.21 | 0.52 |  | 0.13 | 0.34 |  | 0.34 | 0.15 |  | -0.12 | 0.27 |  |  |  |  |  |  |
| Mineralocorticoid:  Glucocorticoid Ratio | | 0.66 | 0.55 |  | 0.06 | 0.70 |  | 0.50 | 0.40 |  | 0.08 | 0.35 |  |  |  |  |  |  |
| SMAD specific E3 ubiquitin-ligase 3 | | 0.29 | 0.29 |  | 0.08 | 0.40 |  | 0.29 | 0.21 |  | -0.34 | 0.35 |  |  |  |  |  |  |
|  |  | **Young** | | | | | | | | | | |  |  |  |  |  |  |
|  |  | **Male** | | | | |  | **Female** | | | | |  |  |  |  |  |  |
|  |  | **High** | |  | **Low** | |  | **High** | |  | **Low** | |  |  |  |  |  |  |
|  |  | ***M*** | ***SD*** |  | ***M*** | ***SD*** |  | ***M*** | ***SD*** |  | ***M*** | ***SD*** |  |  |  |  |  |  |
| Corticotropin-releasing factor | | 0.78 | 0.24 |  | 0.25 | 0.44 |  | -0.02 | 0.66 |  | -0.36 | 0.63 |  |  |  |  |  |  |
| Glucocorticoid receptor alpha | | 0.16 | 0.12 |  | 0.03 | 0.24 |  | -0.12 | 0.28 |  | 0.10 | 0.12 |  |  |  |  |  |  |
| Mineralocorticoid receptor | | 0.51 | 0.14 |  | 0.29 | 0.31 |  | 0.32 | 0.20 |  | -0.29 | 0.56 |  |  |  |  |  |  |
| Mineralocorticoid:  Glucocorticoid Ratio | | 0.35 | 0.03 |  | 0.25 | 0.15 |  | 0.44 | 0.23 |  | -0.39 | 0.49 |  |  |  |  |  |  |
| SMAD specific E3 ubiquitin-ligase 3 | | 0.37 | 0.29 |  | 0.16 | 0.29 |  | -0.12 | 0.12 |  | 0.09 | 0.17 |  |  |  |  |  |  |

**Supplementary Table 3**. ANOVA molecular data results for main effects and interactions

| **Main effects:** | **Age** | | | |  | **Sex** | | | |  | **Stress reactivity** | | | |
| --- | --- | --- | --- | --- | --- | --- | --- | --- | --- | --- | --- | --- | --- | --- |
|  | ***df*** | **F** | ***p*** | **η_p_2** |  | ***df*** | **F** | ***p*** | **η_p_2** |  | ***df*** | **F** | ***p*** | **η_p_2** |
| Heart Telomere Length | **1,23** | **52.91** | **<.001***** | **0.697** |  | 1,23 | 1.17 | 0.292 | 0.048 |  | 1,23 | 3.46 | 0.076 | 0.131 |
| Brain Telomere Length | **1,24** | **11.71** | **0.002**** | **0.328** |  | 1,24 | 0.02 | 0.900 | 0.001 |  | 1,24 | 0.94 | 0.342 | 0.038 |
| Corticotropin-releasing factor | 1,24 | 1.15 | 0.294 | 0.046 |  | **1,24** | **11.87** | **0.002**** | **0.331** |  | **1,24** | **9.20** | **0.006**** | **0.277** |
| Glucocorticoid receptor alpha | 1,24 | 2.16 | 0.155 | 0.082 |  | 1,24 | 0.10 | 0.754 | 0.004 |  | 1,24 | 0.84 | 0.368 | 0.034 |
| Mineralocorticoid receptor | 1,24 | 0.33 | 0.572 | 0.014 |  | 1,24 | 3.35 | 0.080 | 0.122 |  | **1,24** | **8.18** | **0.009**** | **0.254** |
| Mineralocorticoid:  Glucocorticoid Ratio | 1,24 | 1.18 | 0.289 | 0.047 |  | 1,24 | 1.37 | 0.254 | 0.054 |  | **1,24** | **11.04** | **0.003**** | **0.315** |
| SMAD specific E3 ubiquitin-ligase 3 | 1,24 | 0.22 | 0.640 | 0.009 |  | **1,24** | **6.16** | **0.020*** | **0.204** |  | **1,24** | **4.38** | **0.047*** | **0.154** |
| **Two-way interactions:** | **Age and sex** | | | |  | **Age and stress reactivity** | | | |  | **Sex and stress reactivity** | | | |
|  | ***df*** | **F** | ***p*** | **η_p_2** |  | ***df*** | **F** | ***p*** | **η_p_2** |  | ***df*** | **F** | ***p*** | **η_p_2** |
| Heart Telomere Length | 1,23 | 1.86 | 0.186 | 0.075 |  | **1,23** | **10.71** | **0.003**** | **0.318** |  | 1,23 | 0.55 | 0.465 | 0.023 |
| Brain Telomere Length | 1,24 | 0.20 | 0.656 | 0.008 |  | 1,24 | 0.06 | 0.805 | 0.003 |  | 1,24 | 0.91 | 0.349 | 0.037 |
| Corticotropin-releasing factor | 1,24 | 0.57 | 0.458 | 0.023 |  | 1,24 | 0.18 | 0.672 | 0.008 |  | 1,24 | 0.28 | 0.600 | 0.012 |
| Glucocorticoid receptor alpha | 1,24 | 0.13 | 0.718 | 0.006 |  | 1,24 | 0.38 | 0.543 | 0.016 |  | 1,24 | 0.09 | 0.762 | 0.004 |
| Mineralocorticoid receptor | 1,24 | 1.76 | 0.197 | 0.068 |  | 1,24 | 0.36 | 0.555 | 0.015 |  | 1,24 | 2.48 | 0.129 | 0.094 |
| Mineralocorticoid:  Glucocorticoid Ratio | 1,24 | 0.50 | 0.488 | 0.020 |  | 1,24 | 0.03 | 0.873 | 0.001 |  | 1,24 | 0.94 | 0.342 | 0.038 |
| SMAD specific E3 ubiquitin-ligase 3 | 1,24 | 0.14 | 0.713 | 0.006 |  | **1,24** | **4.67** | **0.041*** | **0.163** |  | 1,24 | 0.00 | 0.983 | 0.000 |
| **Three-way interaction:** | **Age, sex and stress reactivity** | | | |  |  | | | | | | | | |
|  | ***df*** | **F** | ***p*** | **η_p_2** |  |
| Heart Telomere Length | 1,23 | 0.01 | 0.916 | 0.000 |  |
| Brain Telomere Length | 1,24 | 2.23 | 0.149 | 0.085 |  |
| Corticotropin-releasing factor | 1,24 | 0.00 | 0.986 | 0.000 |  |
| Glucocorticoid receptor alpha | 1,24 | 2.05 | 0.165 | 0.079 |  |
| Mineralocorticoid receptor | 1,24 | 0.00 | 0.976 | 0.000 |  |
| Mineralocorticoid:  Glucocorticoid Ratio | 1,24 | 2.38 | 0.136 | 0.090 |  |
| SMAD specific E3 ubiquitin-ligase 3 | **1,24** | **4.46** | **0.045*** | **0.157** |  |
| *Significant effects are boldfaced.* |  |  |  |  |  |  |  |  |  |  |  |  |  |  |

**Supplementary table 4**. List of primers used for qPCR analysis

| **Gene name (purpose)** | **Primer name** | **Accession number** | **Sequence (5’ to 3’)** | **Product size (bp)** | **Efficiency (%)** | **Source** |
| --- | --- | --- | --- | --- | --- | --- |
| **Telomere** | Tel1 | N/A | GGTTTTTGAGGGTGAGGGTGAGGGTGAGGGTGAGGGT | 76 | N/A | <https://doi.org/10.1093/nar/30.10.e47> |
|  | Tel2 |  | TCCCGACTATCCCTATCCCTATCCCTATCCCTATCCCTA |  |  |  |
| **cFos (single copy gene)** | cFos-forward | NM_205569.1 | CAGCTCCACCACAGTGAAGA | 176 | 79 | <https://doi.org/10.1371/journal.pone.0086176> |
|  | cFos-reverse |  | GCTCCAGGTCAGTGTTAGCC |  |  |  |
| **corticotropin-releasing factor (HPI axis)** | crf-zf_qPCR-forward | NM_001007379.1 | CGAGACATCCCAGTATCCAAAAAG | 60 | 111 | <https://doi.org/10.1371/journal.pone.0175420.t001> |
|  | crf-zf_qPCR-reverse |  | TCCAACAGACGCTGCGTTAA |  |  |  |
| **mineralocorticoid receptor (HPI axis)** | mr-zf_qPCR-forward | NM_001100403 | CTTCCAGGTTTCCGCAGTCTAC | 75 | 105 | <https://doi.org/10.1371/journal.pone.0175420.t003> |
|  | mr-zf_qPCR-reverse |  | GGAGGAGAGACACATCCAGGAAT |  |  |  |
| **glucocorticoid receptor α (HPI axis)** | gra-zf_qPCR-forward | NM_001020711.3 | ACTCCATGCACGACTTGGTG | 90 | 92 | <https://doi.org/10.1371/journal.pone.0175420.t004> |
|  | gra-zf_qPCR-reverse |  | GCATTTCGGGAAACTCCACG |  |  |  |
| **SMAD Specific E3 Ubiquitin Protein Ligase** | smurf2-zf_qPCR_forward | NM_001114426.1 | TCAGCCTGGATAAGGGTCAAGG | 100 | 105 | Own produced |
|  | smurf2-zf_qPCR_reverse |  | TCACCAAGTTCTTAGCGCACAG |  |  |  |
| **ribosomal protein L13a (housekeeping)** | Rpl-F | NM_212784.1 | TCTGGAGGACTGTAAGAGGTATGC | 148 | 80 | <https://doi.org/10.3389/fnbeh.2015.00271> |
|  | Rpl-R |  | AGACGCACAATCTTGAGAGCAG |  |  |  |
| **actin, beta 2 (housekeeping)** | actin-F | NM_181601.5 | CGAGCTGTCTTCCCATCCA | 86 | 95 | <https://doi.org/10.3389/fnbeh.2015.00271> |
|  | actin-R |  | TCACCAACGTAGCTGTCTTTCTG |  |  |  |
